# Revisiting operons: an analysis of the landscape of transcriptional units in E. coli

**DOI:** 10.1186/s12859-015-0805-8

**Published:** 2015-11-04

**Authors:** Xizeng Mao, Qin Ma, Bingqiang Liu, Xin Chen, Hanyuan Zhang, Ying Xu

**Affiliations:** Computational Systems Biology Lab, Department of Biochemistry and Molecular Biology, and Institute of Bioinformatics, University of Georgia, Athens, USA; BioEnergy Research Center (BESC), Athens, GA USA; College of Computer Sciences and Technology, Changchun, Jilin China; School of Public Health, Jilin University, Changchun, Jilin China; School of Mathematics, Shandong University, Jinan, Shandong China; Present address: MD Anderson Cancer Center, Houston, TX 77054 USA; Present address: Systems Biology and Biomedical Informatics (SBBI) Laboratory University of Nebraska-Lincoln 122B/122C Avery Hall, 1144 T St, Lincoln, NE 68588-0115 USA; Present address: Department of Plant Science, South Dakota State University, Brookings, SD 57006 USA; Present address: BioSNTR, Brookings, SD USA

**Keywords:** Operon, Transcriptional unit, Promoter, Terminator, Bacteria

## Abstract

**Background:**

Bacterial operons are considerably more complex than what were thought. At least their components are dynamically rather than statically defined as previously assumed. Here we present a computational study of the landscape of the transcriptional units (TUs) of *E. coli* K12, revealed by the available genomic and transcriptomic data, providing new understanding about the complexity of TUs as a whole encoded in the genome of *E. coli* K12.

**Results and conclusion:**

Our main findings include that (i) different TUs may overlap with each other by sharing common genes, giving rise to clusters of overlapped TUs (TUCs) along the genomic sequence; (ii) the intergenic regions in front of the first gene of each TU tend to have more conserved sequence motifs than those of the other genes inside the TU, suggesting that TUs each have their own promoters; (iii) the terminators associated with the 3’ ends of TUCs tend to be *Rho*-independent terminators, substantially more often than terminators of TUs that end inside a TUC; and (iv) the functional relatedness of adjacent gene pairs in individual TUs is higher than those in TUCs, suggesting that individual TUs are more basic functional units than TUCs.

**Electronic supplementary material:**

The online version of this article (doi:10.1186/s12859-015-0805-8) contains supplementary material, which is available to authorized users.

## Background

The concept of operon as a transcriptional unit (TU) was first proposed by French scientists Jacob and Monod in 1960 when they were studying the lactose metabolism in *E. coli* [1]. They defined an *operon* as a list of genes that are transcribed in a single polycistronic unit and share the same genetic regulation signals. In their seminal paper [1], Jacob and Monod proposed operons as a model to coordinately transcribe a group of genes arranged in tandem on the same genomic strand, and suggested that all genes in a bacterial cell are controlled by means of operons through a single feedback regulatory mechanism. Since then, operons have been used as the basic transcriptional and functional units in bacterial studies. Such information has been widely applied to derive higher-level functional organizations such as biochemical pathways/networks and regulation systems, which are difficult to derive in eukaryotic organisms.

A widely-held assumption in computational operon prediction has been that operons generally do not overlap [2, 3] although this has never been suggested by Jacob and Monod in their original paper [1]. This assumption allows computational predictions of operons based on sequence-level information alone, and has been popularized through the widely used operon databases such as DBTBS [4], OperonDB [5] and DOOR [6, 7], which were developed based on such an assumption. The rapidly increasing pool of large-scale transcriptomic and proteomic data collected under multiple conditions have clearly shown that this assumption is generally not true [8–10]. Specifically, different subsets of genes in an “operon” may be co-transcribed under different conditions. One such example is that the *pdhR-aceEF-lpd* operon in *E. coli*, consisting of four genes (*pdhR*, *aceE*, *aceF*, *lpd*), has at least three experimentally validated transcriptional units, i.e., the whole operon, (*aceE*, *aceF*) and (*ldp*) under different conditions [11]. The general situation is actually more complex than this as our analysis of large-scale transcriptomic data revealed that generally there may not a mother operon, of which different subsets of its genes are expressed under different conditions; instead the situation tends to be that there are multiple parallel operons, which may overlap but are not subsets of each other, forming a cluster of overlapping TUs along with the genomic sequence. A number of studies aiming to identify TUs revealed by specified RNA-seq data have been published such as [12–16]. We have previously developed a computer program to infer TUs based on strand-specific RNA-seq data [17]. While our initial application was done on *C. thermocellum,* the tool should be generally applicable to any bacteria.

**Fig. 1:**
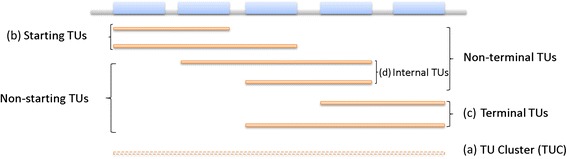
A diagram of TUC and different TU types: (**a**) TUs that span the entire DNA sequence covered by a TUC, referred to as *full TUs*; (**b**) *starting TUs* are the ones that begin with the first gene of their parent TUCs excluding (**a**); (**c**) *terminal TUs* are the ones that end with the last gene of their parent TUCs excluding (**a**); and (**d**) *internal TUs* are the ones that contain neither the first nor the last gene of their parent TUCs (see Fig. 1). TUs of (**b**) and (**d**) are called *non-terminal TUs;* and TUs of (**c**) and (**d**) are *non-starting TUs.* Blue bars represent genes, and each solid orange line represents a TU, and the dashed orange line in the bottom is a TUC

Here we present a computational study of *E. coli* K12 transcriptomic data, aiming to (1) derive as many different TUs as possible based on the available transcriptomic data, and (2) study their genomic locations and regulations. Here a TU is defined as a list of genes, which is transcribed into one RNA molecule under some conditions [18]; hence an “operon” is a TU. To avoid confusions, we use TUs to represent operons as defined by Jacob and Monod, and use “operons” to refer to those computationally predicted and stored in public operon databases. A *TU cluster* (*TUC*) is defined as a maximal set of TUs such that every pair of its TUs are connected with each other, where two TUs are *connected* if they share common genes or they each share common genes with other TUs that are connected. Throughout the paper, a TUC is also referred to as the *parent TUC* of its member TUs. In addition, we have the following definitions: (A) TUs that span the entire DNA sequence covered by a TUC are referred to as *full TUs*; (B) *starting TUs* are the ones that begin with the first gene of their parent TUCs excluding (A); (C) *terminal TUs* are those that end with the last gene of their parent TUCs excluding (A); and (D) *internal TUs* are those that contain neither the first nor the last gene of their parent TUCs. TUs of (B) and (D) are called *non-terminal TUs;* and TUs of (C) and (D) are *non-starting TUs* (see Fig. 1).

Numerous TUs have been experimentally identified in *E. coli* K12. For example, a study by Palsson’s group identified 942 TUs based on genome-scale transcriptomic data collected under four conditions [9]. The RegulonDB contains 842 experimentally validated TUs [19]. We have integrated these datasets plus our own operon prediction in the DOOR database [20] as the currently known TUs of *E. coli K12*, and made a number of discoveries about TUs/TUCs and their regulatory relationships. The most interesting discoveries are that (i) terminators of the terminal TUs tend to be *Rho*-independent terminators, more often than those of the non-terminal TUs; (ii) the intergenic regions in front of the first genes of TUs tend to have more conserved sequence motifs than those of the other genes inside the TUs, suggesting that TUs may each have their own promoters; and (iii) the functional relatedness between adjacent genes within TUs is higher than those within the same TUCs but not the same TUs, indicating that TUs are likely more basic functional units than TUCs. Our analysis programs and the predicted TUCs are available at http://csbl.bmb.uga.edu/~xizeng/research.php?p=TU.

## Results

### Characteristics of TUCs

**Fig. 2:**
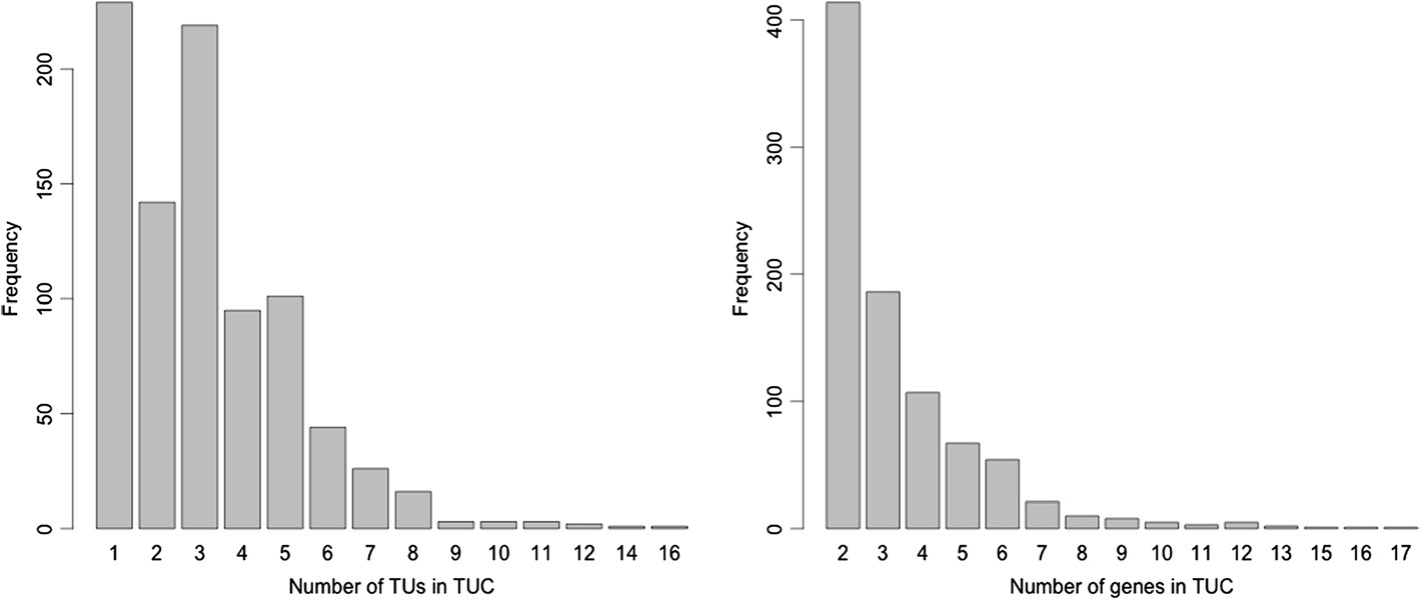
Distributions of the number of genes and the number of TUs per multi-gene TUC across all 885 TUCs

To predict TUCs encoded in the *E. coli* genome, we have integrated the datasets in the Palsson’s paper [9] and RegulonDB database [21] along with *E. coli* operons in our DOOR operon database [7]. This gives rise to a total of 2,227 TUCs, including 1,342 single-gene TUCs and 885 multi-gene TUCs (Additional file 1). Figure 2 shows the size distribution of all the 885 multi-gene TUCs in terms of the number of TUs per TUC, in which 656 (74 %) multi-gene TUCs each have at least two TUs. All the predicted TUCs can be accessed at http://csbl.bmb.uga.edu/~xizeng/research.php?p=TU.

**Fig. 3:**
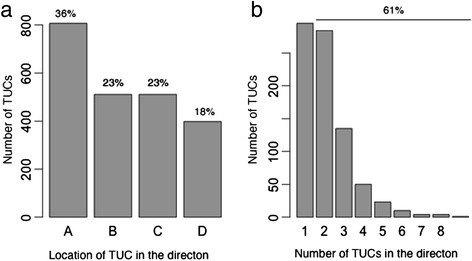
Relations between TUCs and directons in the *E. coli* K12 genome. In (**a**), **A** represents the TUCs matching perfectly with their parent directons; **B** for the TUCs sharing exactly the 5’ boundary genes of their parent directons but; **C** for the TUCs sharing exactly the 3’ boundary genes of their parent directons; and **D** the TUCs located properly inside their parent directons. (**b**) The x-axis represents the number of TUCs per directon and the y-axis represents the number of directons containing *k* TUCs for *k =* 1, …, 8

To study the structure of TUCs in the genome, we have compared them with *directons*, each of which is the maximal set of consecutive genes on the same genomic strand without genes on the opposite strand interrupting the continuity [3, 22]. We intuitively expect that all the TUCs are each contained inside one directon, which proves to be the case based on our analyses. Overall, the *E. coli* genome has 1,318 directons and (at least) 2,227 TUCs. We noted that (a) 807 (36 %) TUCs match perfectly with their parent directons, i.e., sharing the boundary genes at both ends of the directons; 1,022 (46 %) TUCs share exactly one boundary gene of their parent directons; and 398 (18 %) of the TUCs are located properly inside their parent directons (see Fig. 3a); and (b) 806 of the 1,318 (61 %) directons each contain at least two TUCs (see Fig. 3b).

### Non-starting TUs likely have their own promoters

To check whether individual TUs may have their own promoters, we classified all genes in TUCs into three categories: **A**: the set of all 5’-end genes in TUCs; **B**: the set of the 5’-end genes of a TU but not in **A**; and **C**: the set of genes covered by at least one TU but not in (**A** or **B**). We compared the following numbers across the three categories: (i) the number of genes having known and predicted binding sites for transcription factors (TFBS); and (ii) the number of genes having validated promoters downloaded from RegulonDB [21] (see Methods). We found that (i) **B** genes have more known and predicted TFBSs than **C** genes, and less than **A** genes; and (ii) **B** genes have substantially more validated promoters than **C** genes, and less than **A** genes (see Table 1). From these observations, we conclude that TUs are likely to use their own promoters.

**Table 1:** Statistics of 5,430 conserved sequence motifs, 3,307 known plus 2,123 predicted TFBSs, and 3,754 predicted promoters for genes in **A**, **B** and **C**, respectively, with these sets defined above

		*Palsson*			RegulonDB	
	**A** (573)	**B** (456)	**C** (749)	**A** (433)	**B** (445)	**C** (688)
Genes with TFBSs in RegulonDB	233 (39%)	67 (15%)	80 (11%)	178 (41%)	77 (17%)	55 (8%)
Genes with known promoters in RegulonDB	229 (40%)	59 (13%)	47 (6%)	173 (40%)	66 (15%)	29 (4%)

To understand the differences between the **A** genes and the **B** genes, we have examined the lengths of their 5’ upstream inter-genic regions, and compared the average lengths of the inter-genic regions in front of the **A** genes and that of the **B** genes, as well as the average numbers of confidently predicted TFBSs in such regions for the **A** genes *versus* the **B** genes. We found that the average length and the average number of TFBSs are 203 bps and 1.9 for **A** genes, respectively, compared to 101 and 0.5 for the **B** genes in the Palsson dataset; and 195 and 1.8 for the **A** genes *versus* 121 and 0.5 for the **B** genes in RegulonDB. These data suggest that TUs starting with the **A** genes may serve as the default or frequently used TUs compared to the other TUs within each TUC. We then examined the over-represented Gene Ontology (GO) categories by the **A**, **B** and **C** genes, respectively; and found that the **A** genes do not share any of their over-represented GO categories with the (**B** or **C**) genes, while the **B** genes do share some of their over-represented GO categories with the **C** genes, suggesting that non-starting genes in a TU are functionally more relevant with each other. We also noted that these observations are highly consistent between the Palsson set and RegulonDB as summarized in Table 1, providing a cross-validation between the two datasets.

### Non-terminal TUs more likely use *Rho*-dependent terminators

It is known that *E. coli* uses two different mechanisms for transcription termination: *Rho*-independent and *Rho*-dependent termination [23]. *Rho*-dependent termination involves the binding of a *Rho* factor to an mRNA to destabilize the RNA-DNA interaction while *Rho*-independent termination functions by creating an RNA hairpin loop to stop the RNA polymerase [24]. *Rho*-independent terminators can be effectively predicted based on the identification of the conserved RNA hairpin loop, while *Rho*-dependent terminators cannot yet due to the lake of signals known to be associated with them.

To examine if different TUs may have preferences in using either type of termination, we have carried out the following analysis. Using the widely used TranstermHP program [25], we predicted 1,835 *Rho*-independent terminators with confidence score at least 76 for the 4,164 genes of *E. coli*, which is the cutoff for reliable predictions as suggested by the authors of the program. We define the following three sets of genes: **D** as the set of the 3’-end genes of TUCs; **E**: the set of the 3’-end genes of TUs but not in **D**; and **F** the set of all the other genes in TUs but not in (**D** or **E**). We found that (a) **E** uses far fewer *Rho*-independent terminators than **D** percentage-wise; (b) **F** uses a fewer *Rho*-independent terminators than **E**, as detailed in Table 2; and (c) **D**, **E** and **F** genes do not share any of their respectively over-represented GO categories (Additional file 2). These data revealed that (i) TUCs tend to end with *Rho*-independent terminators; (ii) TUs not using the same ends with their parent TUCs use predominantly *Rho*-dependent terminators; and (iii) the predicted *Rho*-independent terminators associated with the **F** genes may represent false predictions, on both the Palsson dataset and the RegulonDB.

**Table 2:** *Rho*-independent terminators for **D**, **E** and **F** genes, as defined above

		Palsson			RegulonDB	
Category	**D** (573)	**E** (335)	**F** (821)	**E** (433)	**E** (359)	**F** (765)
Genes	271 (47%)	46 (14%)	102 (12%)	229 (59%)	70 (19%)	65 (8%)

**Fig. 4:**
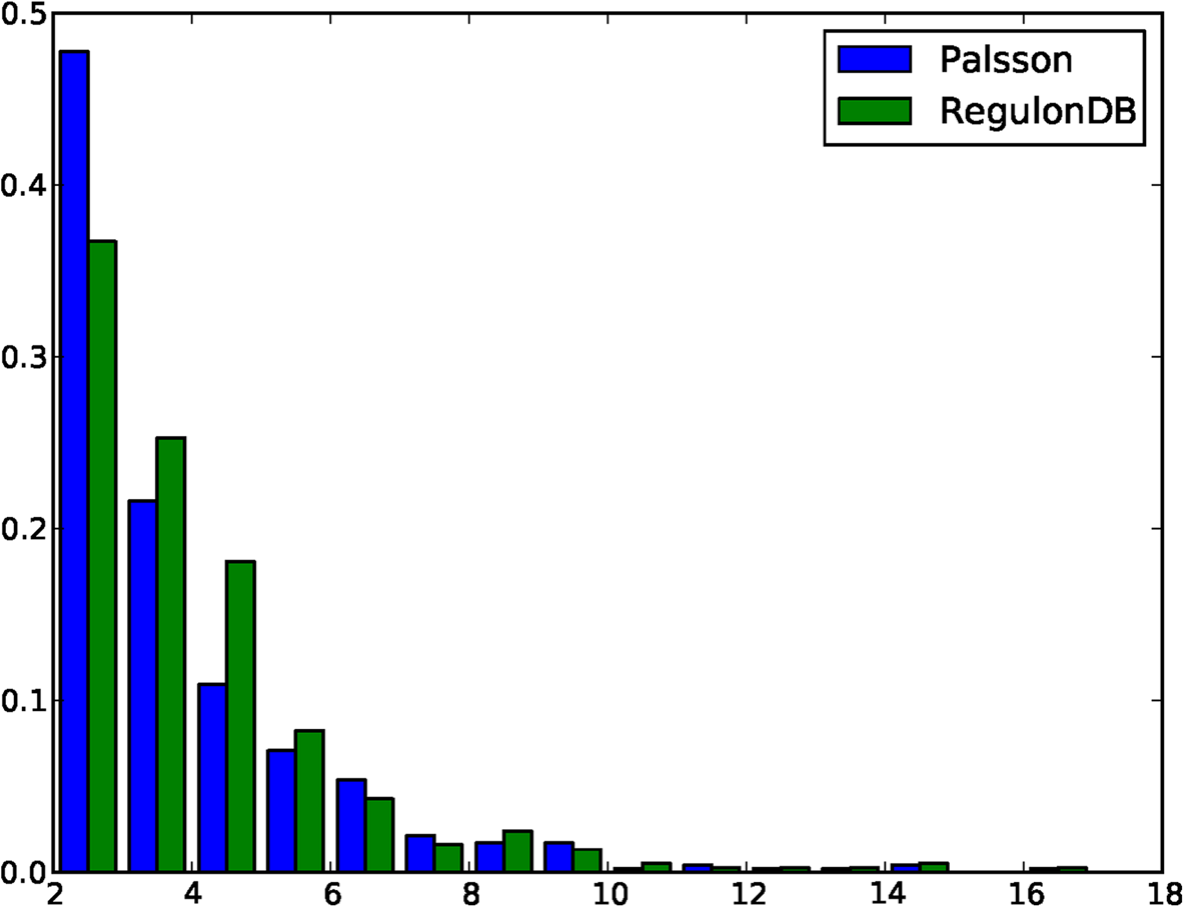
Histograms of TU sizes in the Palsson and RegulonDB datasets

We do note that both the **D** and the **E** sets in Palsson’s dataset are substantially smaller than those in RegulonDB. We suspect that the reason is the segmentation algorithm used Palsson’s study may tend to break a long TU into smaller ones, hence artificially leading to shorter TUCs and hence smaller **E** and **D**. To test if this hypothesis may be true, we have examined the sizes of TUs in both the Palsson set and RegulonDB, and found that the Palsson set indeed has considerably more small TUs consisting of at most two genes, while RegulonDB has more large TUs having 3 to 6 genes, indicating that there is a systematic difference between the sizes of TUs of the two datasets (see details in Fig. 4). This provides a strong supporting evidence to our hypothesis. It is worth noting that we have ignored the larger TUs (size ≥ 7) in above calculation due to their low occurrence frequency (≤5 %).

**Fig. 5:**
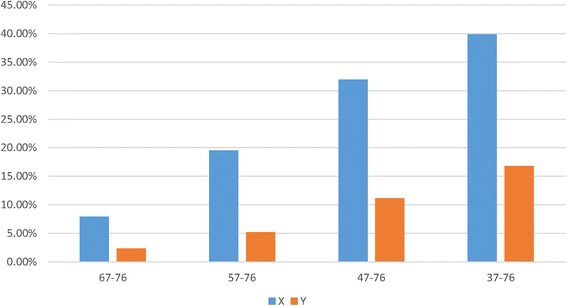
The percentage of genes having *Rho*-independent terminators with confidence score lower than 76, predicted by the TranstermHP program. **X** represents 1,149 3’-end genes of TUCs having *Rho*-independent terminators with confident score no less than 76; and **Y** represents 1,919 non-3’-end genes of TUCs

Another curious issue is that 1,149 of the 2,227 3’-end genes of TUCs, denoted as **X**, are not predicted to end with a *Rho*-independent terminator, suggesting the possibility that an overly stringent cutoff (76 as default) is used by the TranstermHP program. To test this hypothesis, we have re-run the TranstermHP program using lower cutoff values, 37, 47, 57, and 67, on genes in **X** and those non-3’-end genes of TUCs, denoted as **Y**; and found that, percentage-wise**, X** has substantially more *Rho*-independent terminators than **Y** (see details in Fig. 5), which provides a strong supporting evidence to our hypothesis.

### Individual TUs are more basic functional units than TUCs

**Fig. 6:**
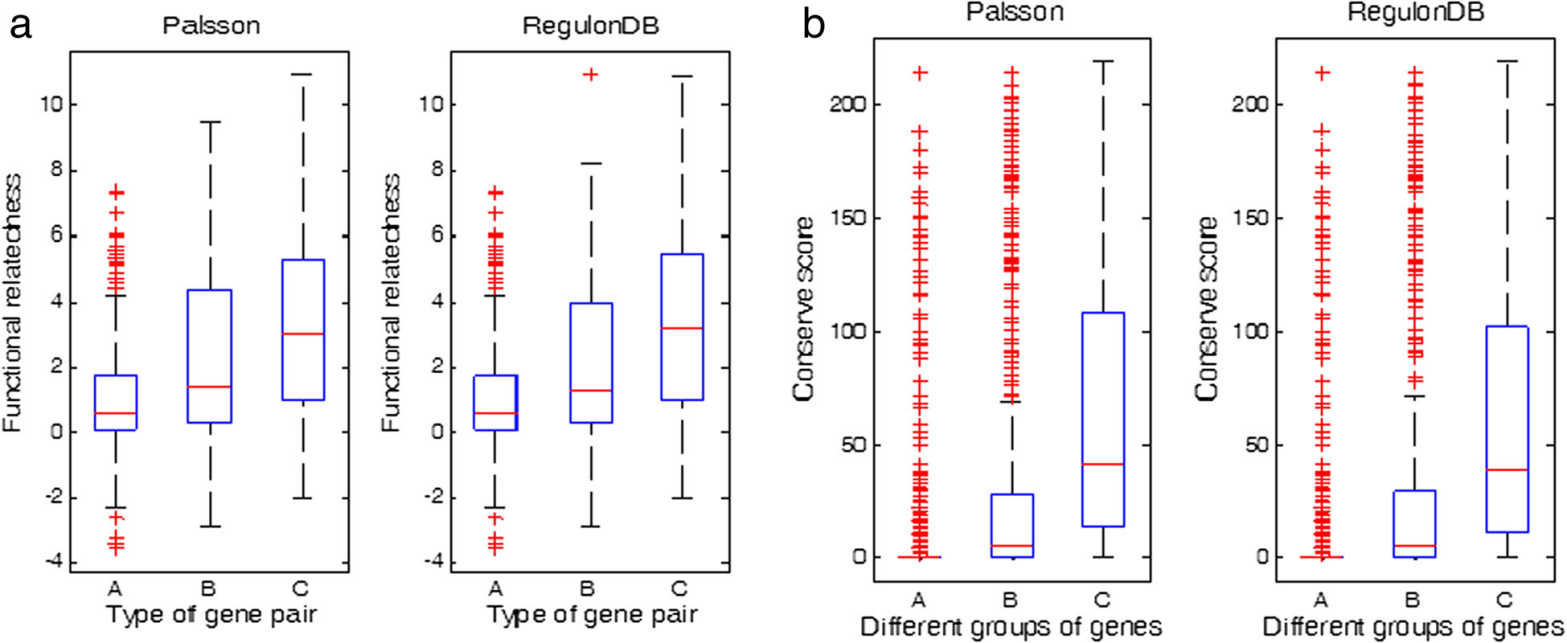
Boxplots of gene functional relatedness and conservation levels for the three types of gene pairs. **a** Gene functional relatedness respectively for the Palsson and RegulonDB data sets; (**b**) gene location conservation levels respectively for the Palsson and RegulonDB data sets

Based on our preliminary analyses, we speculate that TUs are more basic functional units than TUCs. To demonstrate this, we have examined the levels of functional relatedness and the co-occurrence conservation for three types of consecutive gene pairs: **A**: gene pairs each consisting of two adjacent genes in two different TUCs (omitting the cases where both TUCs being single-gene ones); **B**: gene pairs each consisting of a 5’ gene of a TU and the gene in its immediate upstream on the same genomic strand, excluding those in **A**; and **C**: all other gene pairs inside a TU. The functional relatedness of these gene pairs [26] is assessed using a combined phylogenetic profile analysis [27], gene neighborhood analysis [28] and Gene Ontology assignment [29], and the co-occurrence conservation is measured using the number of genomes in which their orthologous genes are adjacent with each other in 216 reference genomes [30] (see Methods). We find from Fig. 6 that the functional relatedness and the co-occurrence conservation level both show clear increasing trend going from the **A** to **B** to the **C** genes, which strongly suggests that TUs likely have served as more basic functional units selected during evolution than TUCs.

## Discussion

Our analyses have shown *Rho*-independent terminators tend to be associated with the end of a TUC, while non-terminal TUs tend to use *Rho*-dependent terminators. This suggests that *Rho*-independent terminators may be associated with the end of a cluster of functionally related genes while *Rho*-dependent terminators are associated with portions of TUCs, which are used under specific conditions that may trigger the release of the *Rho* factors.

It is noteworthy that the TUCs studied here may be smaller than the actual TUCs encoded in the *E. coli* K12 genome as our analysis suggests, as some of the true TUs may not be revealed under the conditions covered by Palsson’s dataset and RegulonDB, which may connect two predicted TUCs into one.

To examine whether the organization of TUCs may be related to chromosomal folding, we have compared the TUCs with the predicted folding domains, called *supercoils*, of the *E. coli* K12 genome, which typically each range from 15Kbps to 100Kbps in length, and the two ends of each supercoil join together through binding with nucleoid associated proteins (NAPs) [31–33] such as H-NS and Fis [34, 35] in a folded chromosome. It has been observed that supercoils may be condition-dependent, i.e., a different set of supercoils may be formed under different conditions [36]. Other than such binding information, no genome-scale supercoil boundary data have been published. We have previously predicted 409 putative supercoils, along with 409 boundary regions in the circular genome of *E. coli K12* based on 527 experimentally validated binding sites of the NAP proteins [32]. We found that 148 out of the 1,078 (606 + 472) TUCs ending with *Rho*-independent terminators have their 3’ ends coincide with (predicted) supercoil boundary regions, and 91 out of the remaining 1,149 TUCs ending with *Rho*-dependent terminators have their 3’-ends coincide with supercoil boundary regions. We have also examined the average gene-expression level of TUCs in the different locations of supercoils under the 466 experimental conditions in the M3D database [37], and found that the TUCs at the supercoil boundaries have higher average gene expression level (with *P*-value 1.1e-4 by the Wilcox test) than those in the middle (Additional file 3). The statistical significance in achieving this level of coincidence for the two cases are 1e-6 and 0.01, respectively, suggesting that supercoil boundaries may play some role in determining the organization of TUCs.

## Conclusion

We have presented a computational study of the landscape of the TUs encoded in the genome of *E. coli* K12, revealed by the available transcriptomic data, and shown new understanding about the organization of TUs as a whole encoded in the genome of *E. coli* K12. Our main findings are: (i) different TUs may overlap with each other by sharing common genes, giving rise to clusters of overlapped TUs, i.e.,TUCs; (ii) the intergenic regions in front of the first genes of TUs tend to have more conserved sequence motifs than those of the other genes inside the TUs, suggesting that TUs each likely have their own promoters; (iii) the terminators associated with the 3’-ends of TUCs tend to be *Rho*-independent terminators, considerably more often than terminators of non-terminal TUs; and (iv) the functional relatedness of adjacent gene pairs within TUs is higher than those in the same TUCs but not in the same TUs, indicating that TUs are likely more basic functional units than TUCs during evolution. To the best of our knowledge, this is the first systemic and large-scale study of the general properties of TUs and TUCs. We anticipate that the knowledge gained here will prove to be useful to scientists who study bacterial genomes, transcription and evolution.

## Methods

### Data

*E. coli* operons used in this study were downloaded from the DOOR operon database at http://csbl.bmb.uga.edu/DOOR/. A total of 2,325 operons are predicted for *E. coli* K12, which includes 884 multi-gene operons covering 2,704 genes and 1,441 single-gene operons. Based on comparisons with experimentally validated operons, the predicted multi-gene operons have an accuracy level at 93.7 % [20].

We have downloaded a dataset of 942 TUs from Palsson’s paper [9] (http://gcrg.ucsd.edu/InSilicoOrganisms/Ecoli) and 842 TUs from the RegulonDB database [19]. The two datasets share 398 common TUs, which is not surprising since TUs are condition-dependent and these two datasets are collected under different conditions. The relatively small overlap between the two sets also suggest that a large number of TUs are not covered by either of these two sets.

2,237 known and 1,770 predicted transcription factor binding sites, 3,754 promoters of *E. coli* are collected from the RegulonDB database [19]. The *TranstermHP* program [25] was used to predict *Rho*-independent terminators in *E. coli*, which has a prediction sensitivity at 89% and specificity at 98% for *B. subtilis* according to the authors of the program. For each TUC without a *Rho*-independent terminator, we consider that it has a *Rho*-dependent terminator.

We downloaded the Gene Ontology categories for *E. coli* from the *org.EcK12.eg.db* R package and used the *GOstats* R package to identify the over-represented categories given a set of genes based on the hypergeometric distribution.

We have predicted 409 supercoil domains and the same number of their boundary regions in the (circular) *E. coli* K12 chromosome [32] using 347 metabolic pathways from EcoCyc [38] and genome-scale gene-expression data collected under 466 conditions in the M3D database [37], based on the following hypothesis: the chromosome of *E. coli* is partitioned into a set of contiguous and independent folding domains under specific growth conditions so that the total number of unfolding of such domains is minimized to make their genes transcriptionally accessible [39]. We then formulated the domain boundary prediction problem as a genome-partition optimization problem and solved it using a dynamic programming approach [32].

### Identification of TU clusters

We have used the two sets of TUs described in Introduction and the 2,325 predicted operons in the DOOR database to predict the TUCs. Overall 4,139 distinct TUs are considered here. We represent each TU as a vertex in a graph, a pair of TUs is connected by an un-weighted edge if they overlap, and each TU Cluster as a maximal connected component. We thus identify each maximal connected component in a graph as a TUC using an in-house Perl script that is accessible on the web page http://csbl.bmb.uga.edu/~xizeng/research.php?p=TU.

### Analysis of functional relatedness of gene pairs

The functional relatedness of gene pairs are accessed from [26], which incorporates phylogenetic profile analysis [27], gene neighborhood analysis [28] and Gene Ontology assignment [29]. Meanwhile, the co-occurrence conservation level of a gene pair is measured by the number of species in which their orthologous genes are adjacent in a list of 216 reference genomes, which are selected within the same phylum but in different genus of *E. coli*, called reference species (Released on 2011-11-01, NCBI). In each genus, we selected the largest genome to avoid potential selection bias in comparative genomics studies [40]. The GOST program [41] is used to identify the orthologous genes of each *E. coli* gene across the 216 reference genomes.

## Additional files

Additional file 1:
**The identified TUCs and their properties and characteristics.** (XLSX 258 kb) Additional file 2:
**The GO functional enrichment analysis.** (XLSX 99 kb) Additional file 3: Figure S1. The comparison of expression values between boundary genes and internal genes. (DOCX 101 kb)

## Abbreviations

DBTBS: database of transcriptional regulation in *B. subtilis*
DOOR: database of prokaryotic operons
*E. coli*: Escherichia coli
EcoCyc: a comprehensive database resource for Escherichia coli
GOST: global optimization strategy
NAP: nucleoid associated proteins
NCBI: National Center for Biotechnology Information
RegulonDB: regulon database
TU: transcriptional unit
TUC: transcriptional unit cluster
