# Supplementary material for: Revisiting operons: an analysis of the landscape of transcriptional units in E. coli
Source: BMC Bioinformatics. 2015 Nov 4;16:356. doi: 10.1186/s12859-015-0805-8 (PMC4634151; doi:10.1186/s12859-015-0805-8)
Supplement: Additional file 3: Figure S1. — The comparison of expression values between boundary genes and internal genes. (DOCX 101 kb) [file 12859_2015_805_MOESM3_ESM.docx]

To study the comparison of gene/TUC expression values in terms of their different locations in the chromosomal folding domains, we have carried out the following analysis. Firstly, we selected the TUCs located at the boundaries and in the middle of each of the 409 predicted supercoil (do not consider supercoils containing less than five TUCs), containing 289 and 285 genes, respectively. These two sets of genes are so-called *boundary genes* and *internal genes*. Then we extracted their expression values from the M3D database, where we can found the normalized genome-scale expression values collected under 466 conditions. For each gene, we use the average value under all the 466 conditions as its expression value to be compared in the following. The comparison results in Figure 1 showcase that the boundary genes have a higher expression level than the internal genes, with a *p*-value of Wilcoxon test as 1.1e-4. And this result is consistent with the science paper mentioned by the first reviewer.





**Figure 1**: The comparison of expression values between boundary genes and internal genes.
